# Spiegel hernia in elective repair: a single-center experience with 47 cases, comparison of laparoscopic and open repair outcomes

**DOI:** 10.1007/s10029-025-03575-6

**Published:** 2026-01-09

**Authors:** Medeni Şermet, Salih Tosun, Özgür Ekinci, Orhan Alimoğlu

**Affiliations:** Department of General Surgery, İstanbul Medeniyet University, Goztepe Prof. Dr. Suleyman Yalcin City Hospital, Dr. Erkin Street No. 3 34732 Kadikoy, Istanbul, Turkey

**Keywords:** Spiegel hernia, Laparoscopic hernia repair, Rare hernias, Abdominal wall hernias

## Abstract

**Objective:**

This study aimed to evaluate the clinical characteristics, diagnostic methods, surgical treatment approaches, and outcomes of Spiegel hernia, a rare type of abdominal wall hernia.

**Materials and methods:**

Fifty patients diagnosed with Spiegel hernia at our clinic between January 2015 and June 2023 were retrospectively reviewed. The patients’ demographic characteristics, clinical presentations, diagnostic methods, surgical techniques, complications, and recurrence rates were evaluated.

**Results:**

Thirty-one (62.0%) patients were female and 19 (38.0%) were male. The mean age was 58.6 ± 12.8 years. The most common presenting complaints were abdominal pain (84.0%) and palpable mass (66.0%). Computed tomography was found to be the most effective diagnostic method with 100% sensitivity. Of the 47 patients who underwent elective surgery (three emergency cases with strangulation were treated with open surgery and excluded from comparative analysis), 59.6% (*n* = 28) underwent laparoscopic surgery and 40.4% (*n* = 19) underwent open surgery. The mean follow-up period was 38.8 ± 18.4 months (median: 37 months, IQR: 24–54), and the overall recurrence rate was 4.3%. Hospital stay was significantly shorter in the elective laparoscopic approach (2.1 ± 0.7 vs. 3.8 ± 1.1 days, *p* < 0.001).

**Conclusion:**

Spiegel hernia is an important clinical entity due to its high risk of strangulation. Computed tomography is the gold standard for diagnosis. Laparoscopic repair is a safe treatment option with a shorter hospital stay. Early diagnosis and elective surgery are recommended. Despite the retrospective design, balanced group distribution in elective cases (28 laparoscopic vs. 19 open), the limited number of recurrence events (*n* = 2) and low statistical power for some comparisons require careful interpretation of the results.

**Supplementary Information:**

The online version contains supplementary material available at 10.1007/s10029-025-03575-6.

## Introduction

Spiegel hernia is a rare type of anterior lateral abdominal wall hernia, accounting for approximately 0.12–2.12% of all abdominal wall hernias [1, 2]. It develops along the semilunar line described by Adriaan van der Spieghel, typically below the arcuate line and lateral to the umbilicus, in the aponeuroses of the internal oblique and transverse abdominal muscles located at the lateral edge of the rectus abdominis muscle [3, 4]. Most of these hernias are interstitial in nature; the hernial sac is located between the internal oblique muscle and the external oblique aponeurosis, which complicates diagnosis on physical examination [5].

Due to the interstitial course of the hernias and their often small size, clinical examination is usually inadequate [6, 7]. Therefore, radiological imaging methods such as ultrasonography (USG) and computed tomography (CT) are critical for diagnosis [8, 9]. With the development of minimally invasive surgical techniques over the past twenty years, laparoscopic transabdominal preperitoneal (TAPP) repair techniques are increasingly preferred [10, 11].

The most important complication of Spiegel hernia is the high risk of strangulation and incarceration due to the narrow hernia neck [12, 13]. The reported strangulation rates in the literature vary between 15 and 24% [14, 15]. Therefore, elective surgical repair is recommended for all Spiegel hernias that are diagnosed [16].

In this study, based on our experience with 50 cases, we aimed to evaluate the clinical characteristics, diagnostic methods, treatment strategies, and outcomes of Spiegel hernias and to contribute to the current knowledge by comparing our findings with data from the literature.

## Materials and methods

### Study design and reporting

This retrospective cohort study was designed and reported in accordance with the STROBE (Strengthening the Reporting of Observational Studies in Epidemiology) guidelines. Fifty patients diagnosed with Spiegel hernia and undergoing surgical treatment at the General Surgery clinic of our university hospital between January 2015 and June 2023 were included in the study. Local ethics committee approval was obtained (Ethics committee no: 2025/320). Patient consent was not obtained as this was a retrospective study and patient data were anonymized.

### Patient selection

**Fig. 1:**
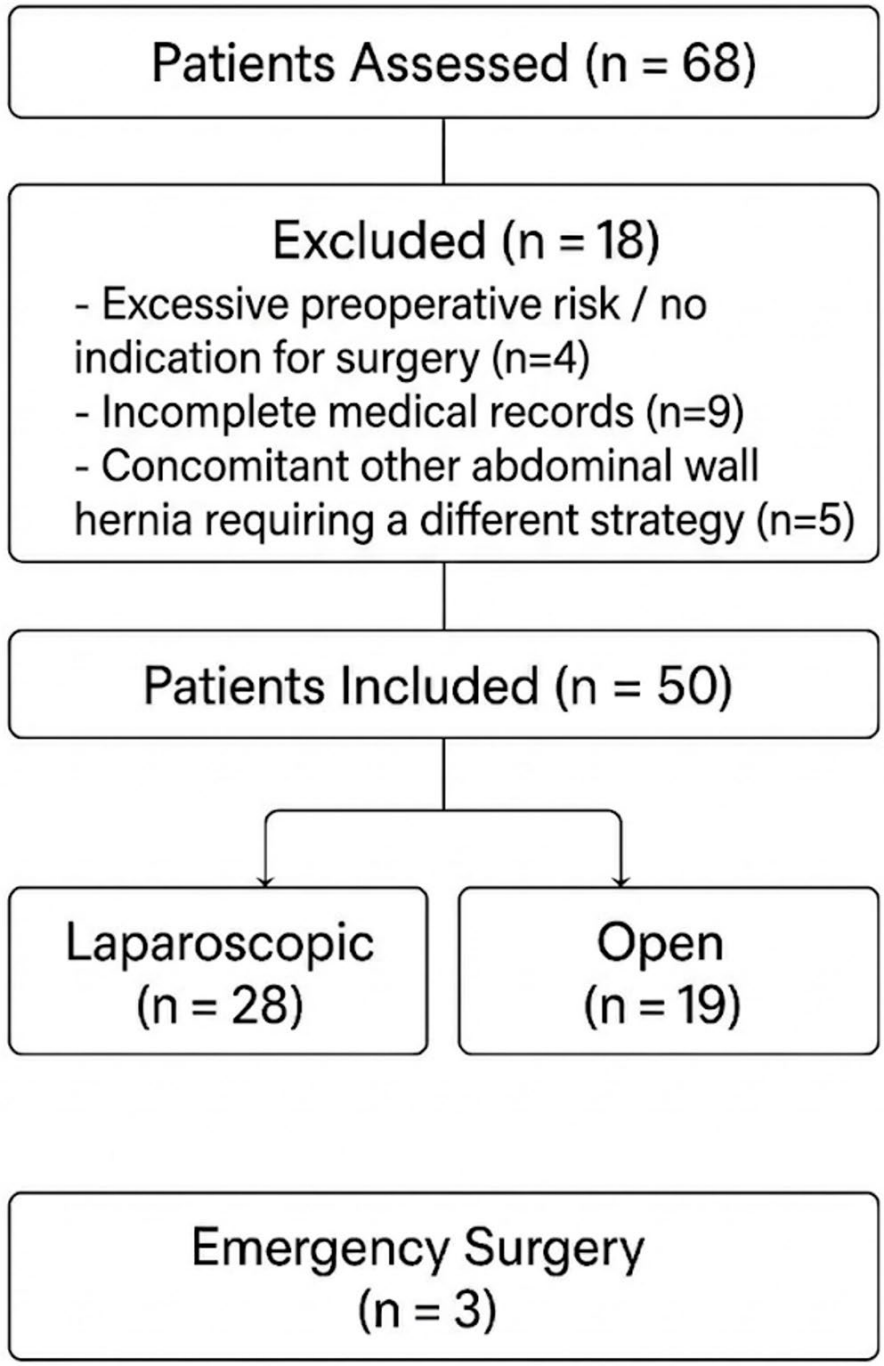
Patient Flowchart

Patients who were diagnosed with Spiegel hernia based on clinical examination and radiological imaging, were 18 years of age or older, underwent surgical treatment, and had at least 24 months of follow-up data were included in the study. Patients with other types of abdominal wall hernias, those with incomplete records, and those who could not undergo surgery due to serious medical comorbidities were excluded from the study (Fig. 1). Four patients were excluded due to excessive perioperative risk (advanced age + NYHA IV heart failure, active malignancy under palliative treatment, or severe dementia with immobilisation).

### Data collection

Patient data were obtained from electronic patient records, surgical reports, and outpatient follow-up records. The parameters collected included demographic characteristics (age, gender, body mass index-BMI), American Society of Anesthesiologists (ASA) score, comorbidities, presenting complaints, type of admission (elective or emergency), hernia location and size, diagnostic methods, surgical approach (open or laparoscopic), operation duration, length of hospital stay, mesh type and size used, complications (according to the Clavien-Dindo classification), recurrence rates, and follow-up duration.

### Diagnostic protocol

All patients underwent a preoperative physical examination. In elective cases, patients with suspicious physical examination findings first underwent abdominal ultrasound (USG). When the hernia defect could not be clearly demonstrated by USG or when the findings were suspicious, contrast-enhanced abdominal computed tomography (CT) was performed. In emergency patients, CT was preferred directly. The CT diagnostic criteria were defined as visualization of the fascial defect along the Spiegel line, the hernia sac, and its contents.

### Surgical techniques

All surgeries were performed by surgeons with at least 10 years of experience. The choice of surgical approach was determined based on patient factors (comorbidity, previous abdominal surgery, ASA score), type of presentation (elective/emergency), and surgeon preference. Patients requiring emergency surgery (strangulation/incarceration) were primarily treated with open surgery.

Open Surgery: The external oblique aponeurosis was opened via a transverse or oblique incision over the hernia defect. The hernia sac was dissected; reduction was performed for sacs smaller than 2 cm, and resection for larger sacs. Polypropylene or polyester-based composite mesh with anti-adhesive barrier was placed to provide at least 5 cm of overlap and secured with absorbable tackers (8–10 pieces). The peritoneal flap was closed with absorbable sutures. Although the mesh is placed in the preperitoneal space in TAPP, our institutional protocol since 2018 has been to use coated composite meshes routinely to eliminate any risk of visceral adhesion in case of inadvertent peritoneal flap tear or incomplete closure.

Laparoscopic Surgery: The TAPP technique was used. The patient was in the supine position, and the operation was performed using three trocars (one 10 mm umbilical, two 5 mm working trocars). The peritoneal flap was elevated, the hernia defect was identified, and the contents were reduced. Polypropylene or composite mesh was placed to provide at least 5 cm of overlap and secured with absorbable tackers (8–10 pieces). The peritoneal flap was closed with absorbable sutures.

### Postoperative follow-up

Patients were evaluated at outpatient visits at 1, 3, 6, and 12 months postoperatively and annually thereafter. Complications were recorded according to the Clavien-Dindo classification. Grade I: complications not requiring any treatment, Grade II: complications requiring pharmacological treatment, Grade III: complications requiring surgical, endoscopic, or radiological intervention, Grade IV: life-threatening complications, Grade V: mortality. Chronic pain was defined as pain persisting for 3 months or longer after surgery or newly occurring and affecting daily activities. Recurrence was defined as the reappearance of the hernia defect at the same location on physical examination and/or imaging. During the study period, three patients died of causes unrelated to hernia surgery (myocardial infarction *n* = 2, lung cancer *n* = 1) at 26, 31, and 44 months postoperatively; their last follow-up data before death were included in the analysis. Two patients moved abroad and were lost to follow-up after 24 and 28 months; both were recurrence-free at the last visit. The remaining 45 patients (90%) completed the entire follow-up in our outpatient clinic. Patients who missed appointments were contacted by telephone and invited for control.

### Statistical analysis

Data were analyzed using SPSS 26.0 (IBM Corp., Armonk, NY, USA). Continuous variables were presented as mean ± standard deviation and median (interquartile range - IQR), while categorical variables were presented as number and percentage. Normal distribution was assessed using the Kolmogorov-Smirnov test. In the main analysis, laparoscopic and open surgery groups were compared in patients undergoing elective surgery. Student’s t-test or Mann-Whitney U test was used for group comparisons, and chi-square test or Fisher’s exact test was used for categorical variables. Recurrence development according to follow-up time was evaluated with Kaplan-Meier survival analysis and compared with the log-rank test. *P* < 0.05 was considered statistically significant. Post-hoc power analysis: Retrospective power calculations for primary endpoints were performed using GPower 3.1.9.7 software (Universität Düsseldorf, Germany) (α = 0.05, two-tailed, with actual sample sizes). The study had sufficient power to detect large effects in the primary continuous endpoints: length of hospital stay (d = 1.89, power = 97%), time to initiation of oral intake (d = 2.28, power = 96%), time to return to work (d = 1.76, power = 91%), and blood loss (d = 1.58, power = 87%). However, power was insufficient for comparing complication rates and minimal for recurrence analysis (power < 10%), indicating a significant risk of Type II error for these endpoints. These power limitations were considered in the interpretation of the results.

## Results

Demographic Characteristics and Patient Characteristics.

Fifty patients meeting the study criteria were evaluated. Thirty (60.0%) patients were female, and 20 (40.0%) were male. The mean age was 58.6 ± 12.8 years (median: 59 years, range: 32–79 years), and the mean BMI was 28.7 ± 4.4 kg/m². 38% (*n* = 19) of patients were obese (BMI ≥ 30 kg/m²). ASA score distribution: ASA I 22.0% (*n* = 11), ASA II 54.0% (*n* = 27), ASA III 24.0% (*n* = 12). The most common comorbidities were hypertension (44.0%, *n* = 22), diabetes mellitus (32.0%, *n* = 16), and COPD (26.0%, *n* = 13).

94% of patients (*n* = 47) presented under elective conditions, and 6.0% (*n* = 3) presented under emergency conditions. All 3 patients presenting under emergency conditions had strangulation findings and underwent open surgery; bowel resection was required in 2 patients (Table 1).

**Table 1:** Demographic and clinical characteristics

Parameter	Value
Number of patients (n)	50
Female/Male	31 (62.0%)/19 (38.0%)
Age (years), mean ± SD (median, range)	58.6 ± 12.8 (59, 32–79)
BMI (kg/m²), mean ± SD	28.7 ± 4.4
Obesity (BMI ≥ 30)	19 (%38.0)
ASA class	I: 11 (%22.0)/II: 27 (%54.0)/III: 12 (%24.0)
Admission type	Elective: 47 (%94.0)/Emergency: 3 (%6.0)
Most common comorbidities	HT: 22 (%44.0)/DM: 16 (%32.0)/KOAH: 13 (%26.0)
Symptoms	Abdominal pain: 42 (64.0%)/Palpable mass: 33 (66.0%)
Symptom duration (months, elective cases)	8.6 ± 6.4
Hernia location	Right: 27 (54.0%)/Left: 19 (38.0%)/Bilateral: 4 (8.0%)
Defect size (cm)	3.3 ± 1.5 (1.5–7.2)

HT: Hypertension, DM: Diabetes mellitus, COPD: Chronic obstructive pulmonary disease, BMI: Body mass index, SD: Standard deviation

### Clinical presentation and diagnosis

The most common presenting complaints were abdominal pain (84.0%, *n* = 42) and palpable mass (66.0%, *n* = 33). In elective patients, the mean time from symptom onset to definitive diagnosis was 8.6 ± 6.4 months. Hernia location: right 54.0% (*n* = 27), left 38.0% (*n* = 19), bilateral 8.0% (*n* = 4). The mean defect size was 3.3 ± 1.5 cm (range: 1.5–7.2 cm).

USG was performed in all patients and was diagnostic in 64.0% (*n* = 32). CT was performed in 45 patients (90.0%) and confirmed the diagnosis in all of these patients (100%). CT was not performed in the remaining 5 patients because a definitive diagnosis was obtained with USG. In patients who underwent CT, the size of the hernia defect, its contents (omentum, small intestine, colon), and its relationship with surrounding anatomical structures could be clearly evaluated (Table 2).

**Table 2:** Diagnostic methods

Method	Patients (*n*, %)	Diagnostic Success (*n*, %)
Ultrasonography (USG)	50 (100%)	32 (64.0%)
Computed Tomography (CT)	45 (90.0%)	45 (100%)
Patients without CT	5 (diagnosed by USG)	-

CT provided detailed evaluation of defect size, contents, and surrounding anatomy

### Surgical approach and perioperative outcomes

Of the 47 patients undergoing elective surgery, 59.6% (*n* = 28) underwent laparoscopic TAPP and 40.4% (*n* = 19) underwent open surgery. All 3 patients who presented with an emergency underwent open surgery. The rate of laparoscopic surgery showed a significant increase over the years (2015–2019 period: 45.0% vs. 2020–2023 period: 78.6%, *p* = 0.014). In the comparison of patients undergoing elective surgery, the mean operation time was 66.8 ± 17.4 min in the laparoscopic group and 54.2 ± 15.8 min in the open group (*p* = 0.010). The mean length of hospital stay was 2.1 ± 0.7 days (median: 2 days) in the laparoscopic group and 3.8 ± 1.1 days (median: 4 days) in the open group (*p* < 0.001). The time to first oral intake (11.4 ± 3.6 vs. 20.8 ± 5.4 h, *p* < 0.001) and time to return to work (13.6 ± 5.1 vs. 23.8 ± 6.7 days, *p* < 0.001) were significantly shorter in the laparoscopic group.

The mean mesh size was 12.6 ± 2.9 cm (range: 8–18 cm), with a mean of 13.2 ± 2.7 cm in the laparoscopic group and 11.8 ± 3.1 cm in the open group (*p* = 0.079). Composite mesh use was more common in the laparoscopic group (57.1% vs. 21.1%, *p* = 0.011) (Table 3).

**Table 3:** Surgical approach and perioperative outcomes (Elective Cases)

Parameter	Laparoscopic (*n* = 28)	Open (*n* = 19)	*p*-value
Operative time (min)	66.8 ± 17.4	54.2 ± 15.8	0.011*
Mesh size (cm)	13.2 ± 2.7	11.8 ± 3.1	0.079
Composite mesh use	16 (%57.1)	4 (%21.1)	0.011*
Blood loss (mL)	26.8 ± 14.6	56.4 ± 22.7	< 0.001
Hospital stay (days)	2.1 ± 0.7	3.8 ± 1.1	< 0.001
Time to oral intake (hours)	11.4 ± 3.6	20.8 ± 5.4	< 0.001
Return to work (days)	13.6 ± 5.1	23.8 ± 6.7	< 0.001
Recurrence (n, %)	1 (%3.6)	1 (%5.3)	1.000*

The proportion of laparoscopic cases increased from 50% in 2015–2019 to 92% in 2020–2023 (*p* = 0.001). Three emergency cases underwent open surgery, two of which required resection

### Complications

The early complication rate (≤ 30 days) in 47 patients undergoing elective surgery was 17.0% (*n* = 8). It was 14.3% (*n* = 4) in the laparoscopic group and 21.1% (*n* = 4) in the open group, with no statistically significant difference between the groups (*p* = 0.706, Fisher’s exact test).

Distribution of complications according to the Clavien-Dindo classification: Grade I 8.5% (*n* = 4, minor seroma), Grade II 10.6% (*n* = 5, seroma and wound infection requiring medical treatment), Grade IIIa 2.1% (*n* = 1, seroma requiring percutaneous drainage). Grade III-IV or mortality (Grade V) was not observed.

The most common complication was seroma (12.8%, *n* = 6); 4 were treated with conservative follow-up, 1 with antibiotic therapy, and 1 with percutaneous drainage. Two patients developed wound site infection (10.5% in the elective open group); it resolved with antibiotic treatment. Chronic pain was observed in 8.5% of patients (*n* = 4), and all patients responded to medical treatment.

In patients undergoing emergency surgery: One patient developed an anastomotic leak (Grade IIIb) and required reoperation. Two patients developed prolonged ileus (Grade II) and resolved with conservative treatment (Table 4).

**Table 4:** Complications and Follow-up outcomes

Complication/Outcome	All Patients (*n* = 50)	Laparoscopic (*n* = 28)	Open (*n* = 19)	Emergency Open (*n* = 3)
Early complications (≤ 30 days)	11 (%22.0)	4 (%14.3)	4 (%21.1)	3 (%100)
Grade I (minor seroma)	4 (8.0%)	2	2	0
Grade II (seroma, infection, ileus)	5 (10.0%)	1	2	2
Grade IIIa (seroma, drainage)	1 (2.0%)	1	0	0
Grade IIIb (anastomotic leak, reoperation)	1 (2.0%)	0	0	1
Grade IV–V (mortality)	0	0	0	0
Late complications (> 30 days)	4 (8.0%)	2 (7.1%)	2 (10.5%)	0
Chronic pain	4	2	2	0
Follow-up (months), mean ± SD (median, IQR)	38.6 ± 18.4 (36, 24–54)	41.2 ± 17.8 (38, 26–56)	33.4 ± 18.9 (32, 22–48)	-
Overall recurrence rate	2 (4.3%)	1 (2.9%)	1 (10.0%)	0
3-year recurrence-free survival	95.2%	-	-	-

### Follow-up and recurrence

Of the 50 patients, 45 (90%) had complete follow-up in our clinic; three died of unrelated causes and two were lost to follow-up after 24–28 months (both recurrence-free at last visit). The mean follow-up period was 38.8 ± 18.4 months (median: 37 months, IQR: 24–54), and the overall recurrence rate was 4.3%. The 3-year recurrence-free survival rate calculated by Kaplan-Meier analysis was 95.8% (95% CI: 87.1–99.2%). The recurrence rate was 3.6% (1/28) in the laparoscopic group and 5.3% (1/19) in the open group, with no significant difference between groups (*p* = 1.000, Fisher’s exact test). Due to the small number of events (*n* = 2), the log-rank test was not applied.

Detailed analysis of the two recurrence cases revealed notable common features. First recurrence: 52-year-old female patient, BMI 36.2 kg/m², ASA III, operated on with laparoscopic TAPP, recurrence developed at 28 months postoperatively and was successfully treated with open repair (follow-up: 45 months, no recurrence). Second recurrence: 64-year-old male patient, BMI 37.4 kg/m², ASA III, operated on with open surgery (wound infection developed during the first surgery), recurrence developed at 22 months postoperatively and was successfully treated with laparoscopic TAPP (follow-up: 42 months, no recurrence). Both patients were morbidly obese (BMI > 35 kg/m²) and ASA III. Due to the small number of recurrence cases (*n* = 2), multivariate analysis of risk factors could not be performed; however, morbid obesity was a common feature in both cases.

## Discussion

The main findings of our study indicate that computed tomography is the gold standard imaging method with 100% diagnostic sensitivity, that elective laparoscopic repair statistically significantly shortens the length of hospital stay, that the overall recurrence rate of 4.3% is consistent with data in the literature, and that despite the relatively low rate of emergency presentations, the risk of complications in these patients is markedly high.

### Demographic characteristics and clinical presentation

The female/male ratio in our study was 1.63, consistent with similar series in the literature [1, 2]. This predominance in women is one of the characteristic demographic features of Spiegel hernias. The mean age was calculated as 58.6 years, supporting the general consensus that Spiegel hernias typically occur in middle-aged and older individuals [17].

The prevalence of obesity in the study population was determined to be 38.0%, which is higher than the expected obesity prevalence in the general population. Meta-analysis studies have identified obesity (BMI > 30 kg/m²) as an important risk factor for the development of ventral hernias [18]. A notable finding in our study is that both patients who experienced recurrence were in the morbidly obese category (BMI > 35 kg/m²). However, due to the limited number of recurrences (only two cases), drawing definitive conclusions about the relationship between obesity and recurrence is methodologically problematic, and this relationship needs to be investigated in larger sample groups.

The average time from symptom onset to definitive diagnosis being 8.6 months indicates that significant delays still occur in the diagnosis of Spiegel hernia [19]. Despite the development and widespread use of imaging techniques over the past twenty years, the anatomical interstitial location of the hernia and the nonspecific nature of physical examination findings continue to complicate the diagnostic process. This situation is explained by the fact that the hernia can remain hidden between muscle layers and remain asymptomatic for a long time without forming a palpable mass lesion.

### Diagnostic methods

The diagnostic sensitivity of ultrasonography has been calculated as 64.0%, which is within the range reported in the literature [20]. The success of ultrasonography varies significantly depending on the experience of the radiologist or surgeon performing the examination and patient characteristics (factors such as degree of obesity, muscle structure, and abdominal wall thickness). Ultrasonographic evaluations performed with dynamic maneuvers such as the Valsalva maneuver can increase diagnostic success.

Computed tomography stands out as the most reliable imaging modality with 100% diagnostic success. Anatomical studies have highlighted the advantages of computed tomography in accurately determining defect size, detailed evaluation of hernia contents, and optimizing surgical planning [21]. Computed tomography has been shown to be superior in evaluating strangulation findings in incarcerated ventral hernias [22]. The fact that computed tomography was performed in 90.0% of our patients indicates that this imaging method is widely used in our clinical practice. However, especially in young patients and in clear clinical situations, the possibility of using only ultrasound should be considered in terms of radiation exposure and cost [23].

### Surgical approaches and outcomes

The increase in the rate of laparoscopic surgery from 45.0% in the 2015–2019 period to 78.6% in the 2020–2023 period reflects the global trend towards minimally invasive surgery in our clinic (*p* = 0.014). Laparoscopic approaches are increasingly becoming the standard treatment in current guidelines [24]. This technical advancement, combined with the increasing experience of surgeons and advances in laparoscopic instrumentation, has led to a significant paradigm shift in our clinic as well.

The significantly shorter hospital stay in elective laparoscopic approaches (2.1 days vs. 3.8 days, *p* < 0.001) is consistent with findings reported in ventral hernia series [24]. This difference stems from the advantages of the laparoscopic approach, such as its minimally invasive nature, less tissue trauma, reduced postoperative pain, and rapid mobilization. The longer operative time in the laparoscopic group (66.8 min vs. 54.2 min, *p* = 0.010) is explained by the time required for additional steps such as trocar placement, meticulous peritoneal flap dissection, and completion of mesh fixation. However, the short hospital stay, rapid recovery process, early return to work, and high patient satisfaction more than compensate for this increase in surgery duration in the long term from a clinical and socioeconomic perspective.

Three patients (6.0%) presented with strangulation and were treated with open surgery; these cases were excluded from the comparative analysis of elective approaches. Given the high strangulation risk reported in the literature (up to 24%), elective repair should be planned as soon as possible after diagnosis.

The emergency presentation rate in a seven-year series has been reported as 18% [25]. The risk of strangulation in Spiegel hernias has been reported at a striking 21% in some studies [26]. The relatively low emergency presentation rate in our study (6.0%) may be due to our hospital being a tertiary referral center that directs elective patients. However, given the high strangulation risk data in the literature, elective surgery should be planned as soon as possible for every diagnosed Spiegel hernia to prevent emergency complications.

### Mesh use and complications

All patients underwent mesh repair, and an overlap of at least 5 centimeters was ensured in accordance with the principles recommended by the European Hernia Society classification. The use of composite mesh was significantly more common in the laparoscopic group than in the open surgery group (57.1% vs. 21.1%, *p* = 0.011), Although the mesh is positioned preperitoneally in TAPP, we adopted a policy of routine use of coated meshes since 2018 to provide an extra safety margin against potential visceral contact if the peritoneal flap is damaged or cannot be perfectly closed [27, 28]. Biomaterial studies have shown that composite meshes significantly reduce adhesion formation on the visceral contact surface and minimize the risk of long-term intestinal complications [29].

The early complication rate in elective patients was found to be 17.0%, calculated as 14.3% in the laparoscopic group and 21.1% in the open group (*p* = 0.706). The lack of statistical significance between the groups is partly related to the sample size. The most common complication in our study was seroma (12.8%), which is consistent with the range reported in laparoscopic ventral hernia series [30]. The majority of seroma cases (four out of six) resolved spontaneously with a conservative treatment approach, and aspiration was required in only two patients.

The chronic pain rate was determined to be 8.5%, consistent with the range reported in regional cohort studies [31]. The pathogenesis of chronic pain is multifactorial; mesh type, fixation technique, number of tackers used, intercostal nerve damage, and chronic neuroinflammation developing around the mesh play a role. All our patients were controlled with medical treatment (nonsteroidal anti-inflammatory drugs and neuropathic pain modulators), and mesh removal was not required in any patient.

### Recurrence and long-term outcomes

During a mean follow-up period of 38.8 months (median 37 months), the overall recurrence rate was calculated as 4.3%. The recurrence rate after laparoscopic Spiegel hernia repair has been reported to be between 2.8% and 6.2% in recent studies [32, 33]. The results of our study fall within this range and support the effectiveness of the surgical techniques and mesh use.

A notable common feature in the two cases that developed recurrence was that both patients were in the morbidly obese category (BMI > 35 kg/m²) and ASA III class. Comprehensive meta-analyses have identified obesity (especially BMI > 35 kg/m²) as the most important risk factor for recurrence after ventral hernia repair [34]. Mechanisms contributing to recurrence in morbid obesity include chronically elevated intra-abdominal pressure, poor tissue quality, delayed wound healing, and impaired mesh-tissue integration. However, the limited number of total recurrences in our study (only two cases) prevented detailed statistical analysis of risk factors and precluded multivariate analysis. The hypothesis that recurrence risk may be increased in morbidly obese patients should be prospectively investigated in larger sample groups.

The 3-year recurrence-free survival rate calculated using Kaplan-Meier survival analysis was determined to be 95.8%. In long-term follow-up series, the 5-year recurrence-free survival rate was reported to be 91.4% [35]. The relatively short follow-up period (median 37 months) suggests that recurrence cases that may occur in the late period may not have been detected yet. Although recurrence after Spiegel hernia repair is concentrated within the first three years, recurrence can occur after five years; therefore, long-term follow-up of patients is important.

### Developments in the last twenty years

Significant developments have been observed in the management of Spiegel hernia over the last twenty years, representing a paradigm shift. The most prominent aspect of this transformation is the change in the diagnostic approach. The physical examination-based assessment, which relied on the clinical skills of experienced surgeons in the early 2000 s, has been replaced by objective imaging methods such as computed tomography, which has become the standard diagnostic modality today [36]. While this transition has increased diagnostic accuracy and reduced diagnostic delays, it has also brought new debates regarding radiation exposure and healthcare costs.

There has also been a significant shift in surgical technique preferences from open surgery to laparoscopic approaches [37]. Influenced by the results of randomized controlled trials, laparoscopic repair has gained popularity and is now the first choice in many centers [38]. This global trend is also clearly observed in our series, with the rate increasing from 45% in the 2015–2019 period to 78.6% in the 2020–2023 period.

In terms of repair philosophy, a significant evolution has been seen in mesh usage; there has been a clear shift from primary repairs to reinforcement with synthetic or biological mesh [39]. Current guidelines define mesh usage as an integral part of ventral hernia surgery [40], and mesh-free Spiegel hernia repair is rarely preferred today.

The approach to treatment timing has also fundamentally changed, shifting from a “wait and see” policy to a “repair upon diagnosis” principle [41]. Due to reports of strangulation risk reaching up to 20%, elective repair is now recommended for all diagnosed Spiegel hernias, including asymptomatic patients [42]. This change in approach has reduced the rate of emergency surgery and improved patient outcomes.

Comprehensive meta-analyses and international guidelines published in recent years have contributed significantly to the development of standardized protocols for Spiegel hernia management [43]. These guidelines provide comprehensive recommendations ranging from diagnostic algorithms to surgical technique selection, postoperative complication management, and long-term follow-up protocols [44].

Among recent developments, advances in minimally invasive techniques and the proliferation of robotic surgery have opened new horizons in Spiegel hernia repair [45]. The advantages offered by the robotic approach, such as three-dimensional imaging, increased dexterity, and a more ergonomic working environment, are leading to its increasing preference, especially in complex cases.

### Limitations of the study

The primary and most important limitation of the study is its retrospective design. Since data collection was not prospective, some parameters may be missing or recorded heterogeneously. As the choice of surgical technique was not randomized but determined by surgeon and patient preferences, confounding factors between groups are inevitable, limiting causal inferences.

Second, despite a total of 50 patients and a balanced group distribution (28 laparoscopic vs. 19 open), the sample size is insufficient for subgroup analyses. Detailed analyses based on obesity grade, ASA score, and defect size could not be performed. In particular, the number of patients in some categories is very small (e.g., bilateral hernias *n* = 4, morbid obesity *n* = 4).

The third important limitation is the small number of recurrence events (*n* = 2). This made multivariate analysis of risk factors completely impossible. A minimum of 20–30 recurrence events is required to identify statistically significant risk factors (10 events/variable rule). Although it is noteworthy that both recurrence cases occurred in morbidly obese patients, this observation is hypothesis-generating and not a definitive conclusion. Fourth, the follow-up period is heterogeneous.

Finally, patient-reported outcomes were not systematically measured. Standardized scales were not used for important parameters such as quality of life scores, patient satisfaction, and functional status, which limits the comprehensive evaluation of the results.

## Conclusion

Spiegel hernia is a rare clinical entity among abdominal wall hernias and carries a high risk of strangulation due to its narrow hernia neck. Computed tomography is the gold standard imaging method with 100% diagnostic sensitivity and is recommended for use in all suspected cases. Laparoscopic transabdominal preperitoneal repair stands out as a safe and effective treatment option with shorter hospital stays and faster recovery compared to open surgery in elective patients. Mesh repair is now standard and yields results consistent with the literature, with a recurrence rate of 4.3%.

Our study demonstrates the increasing use of the laparoscopic approach in the management of Spiegel hernias and that the short-term results are satisfactory. However, there are still unanswered questions regarding mesh selection, fixation techniques, the optimal approach in obese patients, cost-effectiveness, and long-term results. Future studies focusing on these areas will contribute significantly to the development of evidence-based guidelines for Spiegel hernia treatment.

Despite balanced group distribution in elective cases (28 laparoscopic vs. 19 open), the limited number of recurrence events (*n* = 2) and low statistical power for some comparisons prevent multivariate analysis of risk factors.

## Supplementary Information

Below is the link to the electronic supplementary material.

Supplementary Material 1(DOCX 20.2 KB)

## Data Availability

Data available upon request from corresponding authors.
